# 2-(4-Methoxy­phenyl­sulfin­yl)cyclo­hexan-1-one

**DOI:** 10.1107/S1600536809013695

**Published:** 2009-04-18

**Authors:** Julio Zukerman-Schpector, Elisângela Vinhato, Carlos R. Cerqueira Jr, Alessandro Rodrigues, Paulo R. Olivato

**Affiliations:** a Universidade Federal de São Carlos, Laboratório de Cristalografia, Estereodinâmica e Modelagem Molecular, Departamento de Química, São Carlos, SP, Brazil; b Universidade de São Paulo, Conformational Analysis and Electronic Interactions Laboratory, Instituto de Química, São Paulo, SP, Brazil

## Abstract

The cyclo­hexa­none ring in the title compound, C_13_H_16_O_3_S, is in a distorted chair conformation. The intra­molecular S⋯O_carbon­yl_ distance is 2.814 (2) Å. Mol­ecules are connected into a two-dimensional array *via* C—H⋯O contacts involving the carbonyl and sulfinyl O atoms.

## Related literature

For related literature, see: Zukerman-Schpector, da Silva *et al.* (2006 ▶). For structure analysis, see: Cremer & Pople (1975 ▶); Iulek & Zukerman-Schpector (1997 ▶). For details of synthesis, see: Bradscher *et al.* (1954 ▶); Zukerman-Schpector, Maganhi *et al.* (2006 ▶); Drabowicz & Mikolajczyk (1978 ▶).
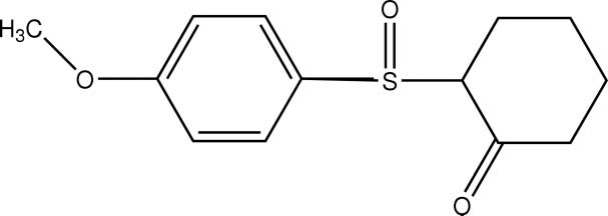


## Experimental

### 

#### Crystal data

C_13_H_16_O_3_S
*M* *_r_* = 252.33
Monoclinic, 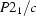

*a* = 11.0510 (4) Å
*b* = 10.0875 (2) Å
*c* = 11.3672 (5) Å
β = 93.886 (2)°
*V* = 1264.27 (8) Å^3^
*Z* = 4
Mo *K*α radiation
μ = 0.25 mm^−1^
*T* = 290 K
0.15 × 0.10 × 0.10 mm

#### Data collection

Bruker APEXII CCD area-detector diffractometer
Absorption correction: none
8283 measured reflections
2872 independent reflections
2508 reflections with *I* > 2σ(*I*)
*R*_int_ = 0.024

#### Refinement

*R*[*F* ^2^ > 2σ(*F* ^2^)] = 0.040
*wR*(*F* ^2^) = 0.113
*S* = 1.06
2872 reflections
155 parameters
H-atom parameters constrained
Δρ_max_ = 0.19 e Å^−3^
Δρ_min_ = −0.23 e Å^−3^

### 

Data collection: *APEX2* (Bruker, 2006 ▶); cell refinement: *SAINT* (Bruker, 2006 ▶); data reduction: *SAINT* and *SADABS* (Bruker, 2006 ▶); program(s) used to solve structure: *SIR97* (Altomare *et al.*, 1999 ▶); program(s) used to refine structure: *SHELXL97* (Sheldrick, 2008 ▶); molecular graphics: *ORTEP-3 for Windows* (Farrugia, 1997 ▶); software used to prepare material for publication: *WinGX* (Farrugia, 1999 ▶), *PARST* (Nardelli, 1995 ▶) and *MarvinSketch* (ChemAxon, 2008 ▶).

## Supplementary Material

Crystal structure: contains datablocks global, I. DOI: 10.1107/S1600536809013695/tk2419sup1.cif

Structure factors: contains datablocks I. DOI: 10.1107/S1600536809013695/tk2419Isup2.hkl

Additional supplementary materials:  crystallographic information; 3D view; checkCIF report

## Figures and Tables

**Table 1:** Hydrogen-bond geometry (Å, °)

*D*—H⋯*A*	*D*—H	H⋯*A*	*D*⋯*A*	*D*—H⋯*A*
C1—H1⋯O2^i^	0.98	2.47	3.257 (2)	137
C3—H3*A*⋯O2^i^	0.97	2.59	3.323 (2)	133
C11—H11⋯O1^ii^	0.93	2.59	3.500 (2)	167

Symmetry codes: (i) 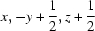; (ii) 
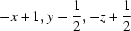
.

**Fig. 1.**
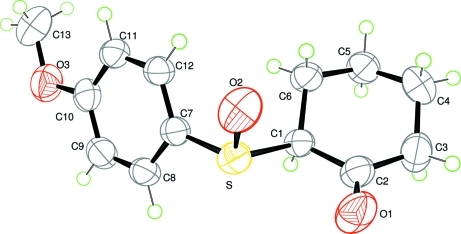
The molecular structure of (I) showing atom labelling scheme and displacement ellipsoids at the 50% probability level (arbitrary spheres for the H atoms).

## supplementary crystallographic information

### Comment

The obtained product, which has stereogenic centres at S and C1, was a 3:1 mixture of the [C1(*R*)S(S)/C1(S)S(*R*)] and [C1(*R*)S(*R*)/C1(S)S(S)] diastereomeric sulfoxides, respectively, as determined from ^1^H NMR spectroscopy. From hexane/ethanol fractional crystallization, the pure [C1(*R*)S(S)/C1(S)S(*R*)] diastereomer, (I), was obtained. The cyclohexanone ring is in a distorted chair conformation as shown by the ring-puckering parameters (Cremer & Pople, 1975; Iulek & Zukerman-Schpector, 1997) q_2_ = 0.143 (2) Å, q_3_ = 0.499 (2) Å, Q = 0.519 (2) Å, φ_2_ = -130.9 (8)°. The methyl moiety is slightly out of the phenyl plane as shown by the C13-O3-C10-C11 torsion angle of 4.9 (2)°. The molecules are linked *via* intermolecular C—H···O interactions involving the carbonyl- and sulfinyl-oxygen atoms into a 2-D array (Table 1).

### Experimental

The starting 2-(4-methoxyphenylthio)cyclohexanone was prepared from the reaction of 2-chlorocyclohexanone and 4-methoxythiophenol as previously reported (Bradscher *et al.* 1954). The sulfoxide 2-[(4-methoxybenzene)sulfinyl]cyclohexanone was prepared by oxidation of 2-(4-methoxyphenylthio)cyclohexanone (Zukerman-Schpector, Maganhi *et al.* 2006; Drabowicz & Mikolajczyk, 1978). A CH_3_OH (10 ml) solution of SeO_2_ (1.23 g, 11.08 mmol) and hydrogen peroxide (30% H_2_O_2_ in aqueous solution; 1.25 ml, 11.08 mmol) was added drop-wise, at 273 K, to a solution of 2-(4-methoxyphenylthio)cyclohexanone (2.62 g, 11.08 mmol) in CH_3_OH (5 ml). The reaction mixture was stirred at 273 K for 2 h and then at room temperature for 2 h. After completion of the reaction, a saturated aqueous NaCl solution (30 ml) was added, the aqueous layer was extracted with CH_2_Cl_2_ (3 *x* 20 ml) and dried over anhydrous Na_2_SO_4_. After solvent evaporation under reduced pressure, 1.39 g (5.5 mmol, yield 50%; m.p. 363–365 K) of the crude 2-[(4-methoxybenzene)sulfinyl]cyclohexanone (I) was obtained. Colourless crystals of (I) were obtained by vapour diffusion from n-hexane/acetone at 298 K.

### Refinement

The H atoms were positioned with idealized geometry using a riding model with C—H = 0.93–0.98 Å, and with *U*_iso_ set to 1.2—1.5 times *U*_eq_(parent atom).

### Crystal data

**Table:**

C_13_H_16_O_3_S	*F*(000) = 536
*M**_r_* = 252.33	*D*_x_ = 1.326 Mg m^−^^3^
Monoclinic, *P*2_1_/*c*	Melting point = 363–364 K
Hall symbol: -P 2ybc	Mo *K*α radiation, λ = 0.71073 Å
*a* = 11.0510 (4) Å	Cell parameters from 5749 reflections
*b* = 10.0875 (2) Å	θ = 1.0–27.5°
*c* = 11.3672 (5) Å	µ = 0.25 mm^−^^1^
β = 93.886 (2)°	*T* = 290 K
*V* = 1264.27 (8) Å^3^	Irregular, colourless
*Z* = 4	0.15 × 0.10 × 0.10 mm

### Data collection

**Table:**

Bruker APEXII CCD area-detector diffractometer	2508 reflections with *I* > 2σ(*I*)
Radiation source: fine-focus sealed tube	*R*_int_ = 0.024
graphite	θ_max_ = 27.5°, θ_min_ = 2.7°
φ and ω scans	*h* = −10→14
8283 measured reflections	*k* = −11→13
2872 independent reflections	*l* = −12→14

### Refinement

**Table:**

Refinement on *F*^2^	Primary atom site location: structure-invariant direct methods
Least-squares matrix: full	Secondary atom site location: difference Fourier map
*R*[*F*^2^ > 2σ(*F*^2^)] = 0.040	Hydrogen site location: inferred from neighbouring sites
*wR*(*F*^2^) = 0.113	H-atom parameters constrained
*S* = 1.06	*w* = 1/[σ^2^(*F*_o_^2^) + (0.055*P*)^2^ + 0.314*P*] where *P* = (*F*_o_^2^ + 2*F*_c_^2^)/3
2872 reflections	(Δ/σ)_max_ < 0.001
155 parameters	Δρ_max_ = 0.19 e Å^−^^3^
0 restraints	Δρ_min_ = −0.23 e Å^−^^3^

### Special details

**Table:**

Geometry. All e.s.d.'s (except the e.s.d. in the dihedral angle between two l.s. planes) are estimated using the full covariance matrix. The cell e.s.d.'s are taken into account individually in the estimation of e.s.d.'s in distances, angles and torsion angles; correlations between e.s.d.'s in cell parameters are only used when they are defined by crystal symmetry. An approximate (isotropic) treatment of cell e.s.d.'s is used for estimating e.s.d.'s involving l.s. planes.
Refinement. Refinement of *F*^2^ against ALL reflections. The weighted *R*-factor *wR* and goodness of fit *S* are based on *F*^2^, conventional *R*-factors *R* are based on *F*, with *F* set to zero for negative *F*^2^. The threshold expression of *F*^2^ > σ(*F*^2^) is used only for calculating *R*-factors(gt) *etc*. and is not relevant to the choice of reflections for refinement. *R*-factors based on *F*^2^ are statistically about twice as large as those based on *F*, and *R*- factors based on ALL data will be even larger.

### Fractional atomic coordinates and isotropic or equivalent isotropic displacement parameters (Å^2^)

**Table:**

	*x*	*y*	*z*	*U*_iso_*/*U*_eq_	
S	0.56721 (4)	0.34807 (4)	0.33452 (3)	0.04990 (15)	
O1	0.79251 (14)	0.37910 (14)	0.46227 (15)	0.0817 (4)	
O2	0.62133 (13)	0.32131 (15)	0.22034 (11)	0.0697 (4)	
O3	0.05306 (12)	0.18455 (16)	0.27603 (13)	0.0739 (4)	
C1	0.63097 (13)	0.22233 (13)	0.43728 (12)	0.0417 (3)	
H1	0.5837	0.2239	0.5072	0.050*	
C2	0.75982 (15)	0.26614 (16)	0.47557 (14)	0.0527 (4)	
C3	0.84104 (17)	0.16348 (19)	0.5352 (2)	0.0672 (5)	
H3A	0.8218	0.1560	0.6169	0.081*	
H3B	0.9245	0.1930	0.5343	0.081*	
C4	0.82992 (16)	0.02795 (18)	0.47825 (18)	0.0634 (5)	
H4A	0.8630	0.0307	0.4014	0.076*	
H4B	0.8762	−0.0360	0.5265	0.076*	
C5	0.69875 (16)	−0.01463 (16)	0.46473 (17)	0.0592 (4)	
H5A	0.6662	−0.0191	0.5418	0.071*	
H5B	0.6933	−0.1024	0.4299	0.071*	
C6	0.62411 (16)	0.08211 (15)	0.38717 (15)	0.0546 (4)	
H6A	0.5402	0.0532	0.3805	0.066*	
H6B	0.6537	0.0823	0.3087	0.066*	
C7	0.41425 (14)	0.29147 (14)	0.31844 (12)	0.0458 (3)	
C8	0.32744 (16)	0.35031 (15)	0.38466 (14)	0.0536 (4)	
H8	0.3502	0.4151	0.4400	0.064*	
C9	0.20829 (17)	0.31257 (19)	0.36820 (16)	0.0599 (4)	
H9	0.1504	0.3521	0.4124	0.072*	
C10	0.17349 (15)	0.21533 (17)	0.28560 (14)	0.0547 (4)	
C11	0.25949 (16)	0.15680 (16)	0.21873 (14)	0.0521 (4)	
H11	0.2368	0.0918	0.1636	0.063*	
C12	0.37932 (15)	0.19623 (15)	0.23499 (13)	0.0490 (3)	
H12	0.4371	0.1585	0.1895	0.059*	
C13	0.0152 (2)	0.0786 (3)	0.1988 (2)	0.0827 (6)	
H13A	0.0360	0.0989	0.1202	0.124*	
H13B	−0.0711	0.0674	0.1995	0.124*	
H13C	0.0550	−0.0018	0.2249	0.124*	

### Atomic displacement parameters (Å^2^)

**Table:**

	*U*^11^	*U*^22^	*U*^33^	*U*^12^	*U*^13^	*U*^23^
S	0.0664 (3)	0.0368 (2)	0.0455 (2)	−0.00370 (15)	−0.00423 (17)	0.00762 (14)
O1	0.0843 (9)	0.0525 (7)	0.1038 (11)	−0.0195 (7)	−0.0274 (8)	0.0100 (7)
O2	0.0803 (9)	0.0861 (9)	0.0433 (6)	−0.0128 (7)	0.0071 (6)	0.0172 (6)
O3	0.0567 (7)	0.0870 (10)	0.0771 (9)	0.0022 (7)	−0.0026 (6)	0.0003 (7)
C1	0.0524 (8)	0.0375 (7)	0.0347 (6)	0.0003 (6)	−0.0003 (5)	0.0025 (5)
C2	0.0593 (9)	0.0462 (8)	0.0517 (8)	−0.0041 (7)	−0.0037 (7)	−0.0007 (7)
C3	0.0525 (9)	0.0611 (11)	0.0856 (13)	−0.0019 (8)	−0.0126 (9)	0.0083 (9)
C4	0.0613 (10)	0.0573 (10)	0.0726 (11)	0.0142 (8)	0.0112 (8)	0.0089 (9)
C5	0.0703 (11)	0.0389 (8)	0.0668 (10)	0.0034 (7)	−0.0073 (8)	0.0049 (7)
C6	0.0688 (10)	0.0370 (7)	0.0560 (9)	0.0013 (7)	−0.0107 (7)	0.0000 (7)
C7	0.0617 (9)	0.0356 (7)	0.0389 (7)	0.0043 (6)	−0.0052 (6)	0.0052 (5)
C8	0.0712 (11)	0.0419 (8)	0.0465 (8)	0.0104 (7)	−0.0038 (7)	−0.0032 (6)
C9	0.0674 (10)	0.0588 (10)	0.0535 (9)	0.0166 (8)	0.0038 (8)	−0.0002 (8)
C10	0.0581 (9)	0.0538 (9)	0.0511 (8)	0.0065 (7)	−0.0050 (7)	0.0094 (7)
C11	0.0645 (10)	0.0448 (8)	0.0455 (8)	0.0035 (7)	−0.0073 (7)	−0.0008 (6)
C12	0.0622 (9)	0.0426 (8)	0.0415 (7)	0.0066 (6)	−0.0013 (6)	−0.0006 (6)
C13	0.0746 (13)	0.0998 (17)	0.0715 (13)	−0.0175 (12)	−0.0124 (10)	0.0023 (12)

### Geometric parameters (Å, °)

**Table:**

S—O2	1.4900 (13)	C5—H5A	0.9700
S—C7	1.7819 (16)	C5—H5B	0.9700
S—C1	1.8325 (14)	C6—H6A	0.9700
O1—C2	1.208 (2)	C6—H6B	0.9700
O3—C10	1.364 (2)	C7—C12	1.386 (2)
O3—C13	1.428 (3)	C7—C8	1.392 (2)
C1—C6	1.525 (2)	C8—C9	1.371 (3)
C1—C2	1.526 (2)	C8—H8	0.9300
C1—H1	0.9800	C9—C10	1.394 (2)
C2—C3	1.501 (2)	C9—H9	0.9300
C3—C4	1.514 (3)	C10—C11	1.388 (2)
C3—H3A	0.9700	C11—C12	1.383 (2)
C3—H3B	0.9700	C11—H11	0.9300
C4—C5	1.510 (3)	C12—H12	0.9300
C4—H4A	0.9700	C13—H13A	0.9600
C4—H4B	0.9700	C13—H13B	0.9600
C5—C6	1.520 (2)	C13—H13C	0.9600
			
O2—S—C7	106.65 (7)	C5—C6—C1	111.56 (13)
O2—S—C1	105.67 (7)	C5—C6—H6A	109.3
C7—S—C1	99.48 (6)	C1—C6—H6A	109.3
C10—O3—C13	117.62 (16)	C5—C6—H6B	109.3
C6—C1—C2	113.39 (13)	C1—C6—H6B	109.3
C6—C1—S	113.37 (10)	H6A—C6—H6B	108.0
C2—C1—S	107.00 (10)	C12—C7—C8	119.70 (15)
C6—C1—H1	107.6	C12—C7—S	120.69 (12)
C2—C1—H1	107.6	C8—C7—S	119.45 (12)
S—C1—H1	107.6	C9—C8—C7	119.90 (15)
O1—C2—C3	122.17 (16)	C9—C8—H8	120.0
O1—C2—C1	121.24 (15)	C7—C8—H8	120.0
C3—C2—C1	116.53 (14)	C8—C9—C10	120.38 (16)
C2—C3—C4	113.65 (16)	C8—C9—H9	119.8
C2—C3—H3A	108.8	C10—C9—H9	119.8
C4—C3—H3A	108.8	O3—C10—C11	124.06 (16)
C2—C3—H3B	108.8	O3—C10—C9	115.92 (16)
C4—C3—H3B	108.8	C11—C10—C9	120.02 (16)
H3A—C3—H3B	107.7	C12—C11—C10	119.29 (15)
C5—C4—C3	110.49 (15)	C12—C11—H11	120.4
C5—C4—H4A	109.6	C10—C11—H11	120.4
C3—C4—H4A	109.6	C11—C12—C7	120.69 (15)
C5—C4—H4B	109.6	C11—C12—H12	119.7
C3—C4—H4B	109.6	C7—C12—H12	119.7
H4A—C4—H4B	108.1	O3—C13—H13A	109.5
C4—C5—C6	110.84 (14)	O3—C13—H13B	109.5
C4—C5—H5A	109.5	H13A—C13—H13B	109.5
C6—C5—H5A	109.5	O3—C13—H13C	109.5
C4—C5—H5B	109.5	H13A—C13—H13C	109.5
C6—C5—H5B	109.5	H13B—C13—H13C	109.5
H5A—C5—H5B	108.1		
			
O2—S—C1—C6	−48.31 (13)	C1—S—C7—C12	−86.59 (13)
C7—S—C1—C6	62.09 (13)	O2—S—C7—C8	−152.43 (12)
O2—S—C1—C2	77.46 (11)	C1—S—C7—C8	97.95 (13)
C7—S—C1—C2	−172.14 (10)	C12—C7—C8—C9	0.9 (2)
C6—C1—C2—O1	143.42 (18)	S—C7—C8—C9	176.38 (12)
S—C1—C2—O1	17.7 (2)	C7—C8—C9—C10	0.1 (2)
C6—C1—C2—C3	−39.3 (2)	C13—O3—C10—C11	4.9 (2)
S—C1—C2—C3	−165.05 (14)	C13—O3—C10—C9	−175.42 (17)
O1—C2—C3—C4	−140.82 (19)	C8—C9—C10—O3	179.84 (15)
C1—C2—C3—C4	41.9 (2)	C8—C9—C10—C11	−0.5 (2)
C2—C3—C4—C5	−51.8 (2)	O3—C10—C11—C12	179.51 (15)
C3—C4—C5—C6	60.5 (2)	C9—C10—C11—C12	−0.1 (2)
C4—C5—C6—C1	−58.4 (2)	C10—C11—C12—C7	1.1 (2)
C2—C1—C6—C5	46.78 (19)	C8—C7—C12—C11	−1.5 (2)
S—C1—C6—C5	169.06 (12)	S—C7—C12—C11	−176.95 (11)
O2—S—C7—C12	23.03 (14)		

### Hydrogen-bond geometry (Å, °)

**Table:**

*D*—H···*A*	*D*—H	H···*A*	*D*···*A*	*D*—H···*A*
C1—H1···O2^i^	0.98	2.47	3.257 (2)	137
C3—H3A···O2^i^	0.97	2.59	3.323 (2)	133
C11—H11···O1^ii^	0.93	2.59	3.500 (2)	167

Symmetry codes: (i) *x*, −*y*+1/2, *z*+1/2; (ii) −*x*+1, *y*−1/2, −*z*+1/2.
